# Magnetoelectric Transducer Designs for Use as Wireless Power Receivers in Wearable and Implantable Applications

**DOI:** 10.3390/ma12030512

**Published:** 2019-02-08

**Authors:** Tyrel Rupp, Binh Duc Truong, Shane Williams, Shad Roundy

**Affiliations:** 1 Space Dynamics Laboratory, Utah State University, Logan, UT 84322, USA; tyrel.rupp@gmail.com; 2 Department of Mechanical Engineering, University of Utah, Salt Lake City, UT 84112, USA; Binh.D.Truong@utah.edu (B.D.T.); shanewill1234@gmail.com (S.W.)

**Keywords:** wireless power transfer, magnetoelectric transducers, piezoelectric transducers, biomedical implants

## Abstract

As the size of biomedical implants and wearable devices becomes smaller, the need for methods to deliver power at higher power densities is growing. The most common method to wirelessly deliver power, inductively coupled coils, suffers from poor power density for very small-sized receiving coils. An alternative strategy is to transmit power wirelessly to magnetoelectric (ME) or mechano-magnetoelectric (MME) receivers, which can operate efficiently at much smaller sizes for a given frequency. This work studies the effectiveness of ME and MME transducers as wireless power receivers for biomedical implants of very small (<2 mm^3^) size. The comparative study clearly demonstrates that under existing safety standards, the ME architecture is able to generate a significantly higher power density than the MME architecture. Analytical models for both types of transducers are developed and validated using centimeter scale devices. The Institute of Electrical and Electronics Engineers (IEEE) and the International Commission on Non-Ionizing Radiation Protection (ICNIRP) standards were applied to the lumped elements models which were then used to optimize device dimensions within a 2 mm^3^ volume. An optimized ME device can produce 21.3 mW/mm^3^ and 31.3 μW/mm^3^ under the IEEE and ICNIRP standards, respectively, which are extremely attractive for a wide range of biomedical implants and wearable devices.

## 1. Introduction

The current explosion of wearable devices has led to increased attention on methods to power them from harvested energy. Implantable Medical Devices (IMDs) might be considered a natural extension of wearable devices especially in the context health related applications. This paper will discuss methods to wirelessly transmit power to IMDs. However, the same methods and devices could easily be applied to wearable devices in order to enhance their usability.

Currently, the most common way to power IMDs is via a direct (wired) external source or a battery implanted along with the IMD. Direct power delivery may cause limitations to patient mobility and creates medical risks associated with passing wires transcutaneously. Batteries help mitigate the problems presented by the direct powering method, however they have finite lifetimes and require periodic replacement. This concern is particularly relevant as the sensing and computation components of IMDs become very small (i.e., 1 mm^3^ or smaller). In light of these concerns, a Wireless Power Transfer (WPT) system would appear to be a promising solution.

Acoustic WPT systems have recently been investigated largely due to the fact that acoustic power has low attenuation in soft tissue and short wavelengths (compared to electromagnetic wavelengths) which increases the efficiency of very small receivers. However, acoustic power transfer systems are complicated by poor transmission through bone and the need for the transmitter to be in direct contact with the skin [1]. For a detailed review of acoustic WPT systems applied to IMDs the reader is referred to [2].

Electromagnetic WPT systems have been widely investigated for use by IMDs. For the purposes of this paper, we classify electromagnetic WPT as either Inductive Power Transfer (IPT) if the coupling is in the near field, or Radio Frequency (RF) if the coupling is in the mid field (i.e., transition region) or far field. Inductive coupling techniques appear to be the most advanced for powering implants. Inductive coupling utilizes a pair of coils that must be physically close and well aligned to allow for the transfer of power. Subsequently, the power transfer is highly dependent on the size, orientation, and distance between the coils [3]. Ultimately, these dependencies make this form of WPT most viable for IMDs (such as pacemakers [4]) where the depth of the implant is relatively shallow and the alignment of the coils can be well controlled.

Unlike acoustic and inductively coupled systems, alignment is not as critical for RF WPT systems because receivers do not need to be tightly coupled to the transmitter. However, as the size of receivers decrease, the operating frequency must increase to a level where tissue tends to absorb and attenuate the transmitted signal [5]. This attenuation is not only inefficient, but it is potentially hazardous because of associated tissue heating. A technology that could make efficient use of low frequency electromagnetic power transfer at a distance could be a significant enabler for very small implantable systems. Magnetoelectric (ME) transducers could be such a technology.

The magnetoelectric effect refers to any type of coupling between electric and magnetic fields found in matter [6]. The ME effect was first demonstrated experimentally in 1960 when Dzyaloshinskii witnessed it in Cr_2_O_3_ [7]. Despite this breakthrough, subsequent research showed that at best the magnetoelectric coefficient for bulk materials such as Cr_2_O_3_ was very low, on the order of 100 mV/(cm·Oe) [7]. This, along with other various complications, kept the materials from being used much in practical applications [6]. Before the ME effect was even observed in bulk materials, Tellegen suggested developing composites that demonstrated a cumulative ME effect [8]. The implication here is that by coupling two separate physical effects (piezoelectric (PE) and magnetostrictive (MS)) in two separate materials an equivalent ME effect could be obtained. In PE materials, the mechanical strain and electric field are coupled. In MS materials, the mechanical strain and magnetic field are coupled. By linking two such materials mechanically, the resulting pseudo ME effect can be demonstrated simply as [9]

(1) $$\mathrm{ME}\;\mathrm{Effect}=\frac{\mathrm{electrical}}{\mathrm{mechanical}}\times \frac{\mathrm{mechanical}}{\mathrm{magnetic}}$$

where the mechanical components in essence cancel out. In 1998 Shin et al. attempted a three-layer laminate composite approach where the MS material was sputtered as a thin film onto a glass substrate which was then bonded onto a PE base. This design has become known in the literature as a unimorph. By applying a voltage to the PE, the bending strain induced into the MS material caused large changes to its magnetic properties [10].

**Figure 1:**
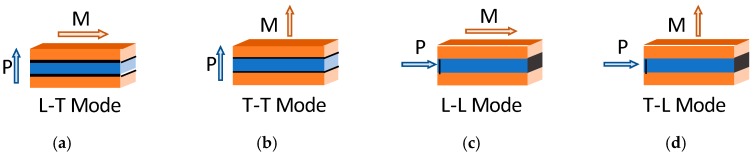
Four bimorph laminate orientation combinations. Orange indicates magnetostrictive (MS) material, blue the piezoelectric (PE) material, and black the location of the PE electrodes. Additionally, the letters in the mode names, T for transverse and L for longitudinal, indicate the orientation of the MS material and PE material, respectively. (**a**): L-T Mode; (**b**) T-T Mode; (**c**) L-L Mode; (**d**) T-L Mode. Compiled from [12,13,14,15].

Building on the ME laminate approach taken by Shin et al., Ryu et al. developed another ME laminate using a sandwich design. This design used silver epoxy to bond a PZT-5A PE disk between two Terfenol-D MS disks. Measuring the magnetoelectric coefficient under various magnetic field strengths yielded values up to 4.68 V/(cm·Oe) [11]. Not only was this an overwhelming improvement to the magnetoelectric coefficient, it brought the ME effect to a point of usefulness. Following closely on the work done by Ryu et al., Dong et al. published a series of papers which have become seminal works for the design and modeling of sandwich, or extensional bimorph, ME transducer designs and configurations [12,13,14,15]. Their work created model subsets for each of the four coupling orientation combinations possible for the PE and MS materials within the laminate structure. These configurations are compiled and shown in Figure 1 and indicate whether the PE and MS materials are poled longitudinally or transversely to the bimorph structure.

Fundamentally, a magnetoelectric transducer is any device that takes energy from the magnetic domain to the electric domain and vice versa. Recently, another type of ME transducer has been proposed. This transducer operates by coupling the moment induced on a ferromagnet by an external magnetic field with a bending PE beam. This is done by anchoring one end of the beam and by mounting the ferromagnet at the tip of the beam oriented perpendicular to the field [16]. To this point, most of the research done on such a device has been for the purpose of energy scavenging and the corresponding models are sparse. Nevertheless the design shows significant promise for WPT [16,17,18]. Publications have yet to use a consistent name for this geometry. It will be referred to in this work simply as a Mechano-Magnetoelectric or MME device.

A significant advantage of magnetoelectric transducers for the use in WPT systems for IMDs is that for a given operating frequency, an efficient receiver can be orders of magnitude smaller than for IPT or RF WPT systems. This occurs because the electromagnetic wave is converted to a mechanical acoustic wave before being transduced to electricity. For a given frequency, acoustic wavelengths are much smaller than electromagnetic wavelengths. O’Handley et al. first suggested utilizing a ME bimorph for transcutaneous power transfer in 2008. Their research showed that in air a 0.1 cm^3^ receiver could generate 2 mW at a distance of 3 cm from a transmitting solenoid [19]. Citing O’Handley et al., Paluszek et al. make cases for how wireless endoscopy, brain imaging, and surgical tools might benefit from the use of ME based WPT [20]. Nonetheless, it would appear that with the exception of some finite element verification work, very little has been done to move the medical research forward [21].

The purpose of this paper is to critically evaluate ME laminates and MME transducers as candidates for wireless power receivers in a WPT system for IMDs. As such, the evaluation incorporates existing medical safety constraints [22,23,24]. Lumped element models for both types of systems are developed and experimentally validated. Using these models, optimal transducer designs are obtained using numerical optimization techniques. These two optimal designs are compared to one another and to the needs of theoretical WPT powered IMDs.

## 2. Materials and Methods

The basic approach taken in this work is to develop lumped element models for each type of transducer, fabricate test transducers using off-the-shelf materials, experimentally validate the models using the test transducers, and numerically optimize designs for a micro-fabricated version of the ME transducers using the validated models. This section will cover the modeling, fabrication of test structures, and experimental methods.

### 2.1. Lumped Element Model for ME Devices

**Figure 2:**
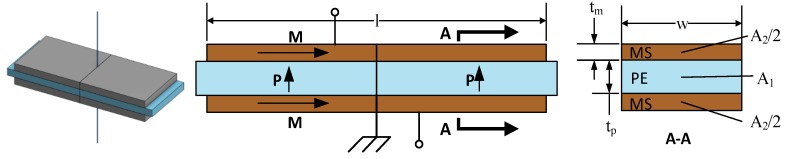
Geometry layout for L-T mode bimorph. Arrows M and P show the magnetization and polarization orientation. Adapted from [13].

**Figure 3:**
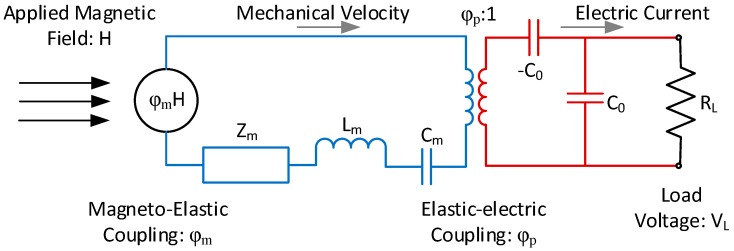
Magnetoelectric equivalent circuit with added load resistor. Adapted from [13].

Dong et al. [14,15] rank the four ME laminate configurations shown in Figure 1 in descending order of the magnetoelectric voltage coefficient as L-L, L-T, T-L, T-T. In practical terms, the L-L and T-L configurations are extremely difficult to fabricate on a small scale. Therefore, the L-T configuration was chosen for further consideration. The laminate is considered to be mounted at the center, as shown in Figure 2, making a longitudinal mode resonator. This structure can be modeled by the equivalent circuit shown in Figure 3, which is a slight alteration of the equivalent circuit developed by Dong et al. in [13]. A load resistor has been added to enable the calculation and optimization of received power.

As shown in Figure 3, the MS transduction is modeled by the magnetoelectric coupling factor, *φ_m_*, which is defined as

(2) φm=A2d33,ms33H [NAm]

where $A_{2}$ is the total cross-sectional area of the MS layers, $d_{33,m}$ is the magneto-elastic or piezomagnetic (PM) coefficient in the longitudinal direction, and $s_{33}^{H}$ is the elastic compliance of the MS material also in the longitudinal direction. When multiplied by the magnetic field level H, $\varphi_{m}$ yields the force caused by the MS layer. Similarly, the PE transduction in the model is defined by the elasto-electric or piezoelectric coupling factor, *φ_p_* which is defined as

(3) $$\varphi_{p}=\frac{wlg_{31,p}}{t_{p}s_{11}^{D}\;{\bar{\beta }}_{p}}\;\left[\frac{N}{V}\right]$$

where w and l are the width and length of laminate, $t_{p}$ is the thickness of the piezoelectric layer, $g_{31,p}$ is the transverse piezoelectric voltage coefficient, $s_{11}^{D}$ is the longitudinal compliance, and ${\bar{\beta }}_{p}$ is the inverse dielectric constant. The piezoelectric coupling is modeled as a transformer in the Lumped Element Model (LEM) and relates the force caused by the MS layer to the voltage of the PE layer.

The electrical capacitance in the circuit, $C_{0}$, is the clamped capacitance of the piezoelectric material, and is defined as

(4) $$C_{0}=\frac{wl}{t_{p}\;{\bar{\beta }}_{p}}\;\left[\frac{N}{V}\right]$$

The value for ${\bar{\beta }}_{p}$, the effective inverse dielectric constant, is calculated by

(5) $${\bar{\beta }}_{p}=\beta_{p}\left(1+\frac{g_{31}^{2}}{s_{11}^{D}\beta_{p}}\right)\;\left[\frac{m}{F}\right]$$

The mechanical damping coefficient, $Z_{m}$, inductance (inertia), $L_{m}$, and capacitance (compliance), $C_{m}$ are defined as

(6) $$Z_{m}=\frac{\pi Z_{0}}{4Q_{m}}\left[\frac{kg}{s}\right]$$

(7) $$L_{m}=\frac{\pi Z_{0}}{4\omega_{s}}\;\left[kg\right]$$

(8) $$C_{m}=\frac{1}{\omega_{s}^{2}L_{m}}\left[\frac{s^{2}}{kg}\right]$$

where $Q_{m}$ is the effective mechanical quality factor for the laminate, $\omega_{s}$ is the fundamental frequency of the laminate, and $Z_{0}$ is the characteristic mechanical impedance of the laminate in the extensional model. These remaining lumped mechanical parameters were derived by Dong et al. [15] by solving the second order equation of motion for the system. The results of this derivation are summarized in Table 1.

A frequency domain circuit analysis on the equivalent circuit of Figure 3 yields the following expression for the effective ME coefficient,

(9) $$\alpha_{me}=\frac{\partial V_{L}}{\partial H}=\left|\beta \frac{\;\varphi_{p}}{j\omega C_{0}+\frac{1}{R_{L}}}\;\frac{\varphi_{m}}{\;Z_{m}+j\omega L_{m}+\frac{1}{j\omega C_{m}}+\varphi_{p}^{2}Z^{\prime }}\right|\left[\frac{V}{A/m}\right]$$

where β≤1 is the ME bias factor, which will be discussed in more depth in the following section, ω is the operating frequency of the magnetic field H, and $Z^{\prime }$ is the impedance of the electrical portion of the circuit of Figure 3 given by

(10) $$Z^{\prime }=\frac{R_{L}}{j\omega C_{0}R_{L}+1}-j\omega C_{0}\left[\Omega \right]$$

One will note that the piezoelectric coupling factor, $\varphi_{p}$ in Equation (9), is not squared as it is in [15]. It appears that this is an error in the reporting of the original model in [15].

The zero-peak load voltage ($V_{L}$) can then be calculated by

(11) $$V_{L}=H_{p}\left|\alpha_{me}\right|\;\left[V\right]$$

where $H_{p}$ is the magnitude of the sinusoidal magnetic field. Finally, the RMS power $\left(P_{RMS}\right)$ is calculated as

(12) $$P_{RMS}=\frac{1}{2}\frac{V_{L}^{2}}{R_{L}}\;\left[W\right]$$

**Figure 4:**
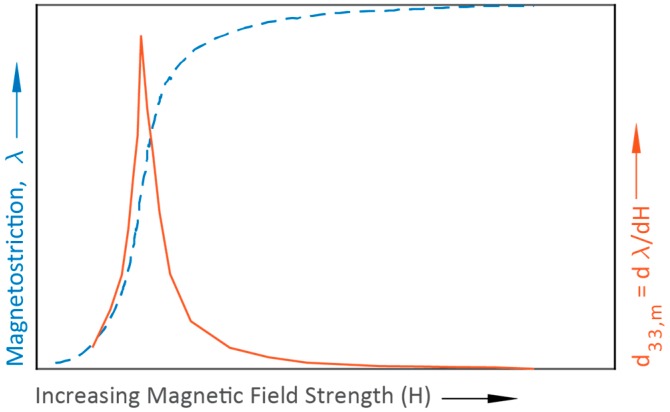
A typical magnetostriction profile and its derivative. Adapted from [25].

This model makes a few assumptions that need to be made explicit. First, the model assumes complete and uniform strain transfer from the MS layer into the PE layer. This implies that the interface joint between the laminates is infinitely stiff and that there is no strain gradient through the thickness of the laminate. Second, the model assumes that materials operate linearly (i.e., material properties are constant). This is probably a good assumption for the material compliance and piezoelectric voltage coefficients. However, magnetostriction is more complicated. The piezomagnetic coefficient is defined as

(13) $$d_{33,m}=\frac{d\lambda }{dH}$$

where λ is the magnetostriction of a given MS material [25]. Simply put, the PM coefficient is the rate of change of magnetostriction with respect to magnetic field. As indicated by Figure 4 magnetostriction is nonlinear and furthermore $d_{33,m}$ is quite low at or near zero magnetic field. An effective transducer will operate near a point where $d_{33,m}$ is at a maximum, which requires that the MS material be biased. Biasing is done by applying a direct current (DC) magnetic field ($H_{DC})$) to effectively move the ME laminate operating point to the maximum piezomagnetic coefficient value. To account for the DC magnetic field bias, which varies tremendously by material, Dong et al. added the variable β to Equation (9). A value of β=1 means the structure is optimally biased; a value of β=0 means the structure is not biased at all [15]. This component of the model has to be evaluated experimentally as the optimal bias varies by geometry, material selection, and mechanical preload. Work has been done to build self-biased ME structures that eliminate the need for biasing, however, that research is still premature and beyond the scope of this work [26].

### 2.2. Lumped Element Model for MME Devices

**Figure 5:**
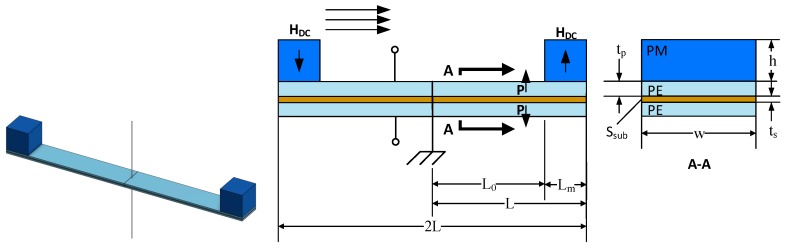
Geometry layout for the double cantilever mechano-magnetoelectric (MME) structure. Arrows marked P indicate PE poling directions and arrows marked H_DC_ indicate the orientation of the permanent magnetic fields.

Figure 5 shows a MME device. The device utilizes a single piezoelectric bending laminate composed of a PE top layer, a structural center layer (S_sub_), and another symmetric PE bottom layer. Strain is induced on the structure by anchoring the bending laminate at the center and adding oppositely oriented permanent magnets at its ends. When a magnetic field is applied along the length of the structure, the beam experiences a pure bending moment.

**Figure 6:**
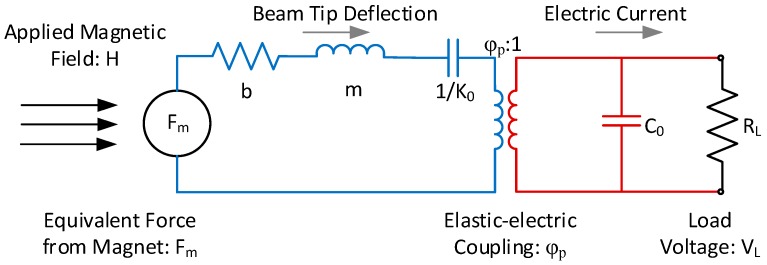
Equivalent circuit model of a double cantilever MME structure.

The equivalent circuit model for the structure in Figure 5 is shown in Figure 6. By inspection it can be seen that the model is fundamentally similar to the ME laminate model and shares the same parameters for the piezoelectric portion of the circuit.

The power output of the MME structure is calculated as

(14) $$P=\frac{1}{2}\Delta K\frac{\omega^{2}\tau }{1+\omega^{2}\tau }{\left|X_{0}\right|}^{2}\;\left[W\right]$$

where

(15) $$\tau =R_{L}C_{0}\;\left[s\right],$$

(16) $$\Delta K=\frac{\varphi_{p}^{2}}{C_{0}}\;\left[\frac{N}{m}\right],$$

(17) $$\varphi_{p}=-\frac{4e_{31}w\left(t_{p}+t_{s}\right)\left[3\left(M+m_{b}\right)L^{2}-3m_{b}L_{0}L+m_{b}L_{0}^{2}\right]}{6\left(M+m_{b}\right)L^{3}-6m_{b}L_{0}L^{2}+2L_{0}^{2}L\left(M+2m_{b}\right)-L_{0}^{3}m_{b}}\;\left[\frac{N}{V}\right],$$

and

(18) $${\left|X_{0}\right|}^{2}=\frac{F_{o}^{2}}{{\left[\omega b+\Delta K\frac{\omega \tau }{1+{\left(\omega \tau \right)}^{2}}\right]}^{2}+{\left[K_{1}-m\omega^{2}-\Delta K\frac{1}{1+{\left(\omega \tau \right)}^{2}}\right]}^{2}}\;\left[m^{2}\right]$$

At optimal load resistance and open-circuit resonance frequency, the optimal average power is stated as

(19) $$P_{AVG}^{opt}=\frac{F_{0}}{4b}M_{1}\left(\sqrt{M_{1}^{2}+1}-M_{1}\right)\;\left[W\right]$$

where

(20) $$M_{1}=\frac{\Delta K}{b\omega_{1}}$$

In this case, the optimal load resistance is calculated as

(21) $$R_{L}^{opt}=\frac{\sqrt{M_{1}^{2}+1}}{\omega_{1C_{0}}}\left[\Omega \right]$$

For the sake of simplicity, the variables and constitutive equations that compose Equations (14)–(21) are summarized in Table 2. One can note that the model for the MME is significantly more convenient than the ME model because there is a closed-form solution for the optimal load and power. A full derivation of Equations (14) though (21) is found in [18]. (Note that unlike [18], Figure 5 neglects the finite length of the center clamp. However, this has no effect on the generality of the model.).

### 2.3. Fabrication of Test Structures

Two ME transducers were built to validate the model. Two material structure combinations were built: a Galfenol and lead zirconate titanate (PZT) laminate and a Metglas 2605SA1 (Metglas^®^ Inc., Conway, SC, USA) and polyvinylidene fluoride (PVDF) laminate. Terfenol-D was avoided due to the difficulties associated with machining a brittle, pyrophoric material.

**Figure 7:**
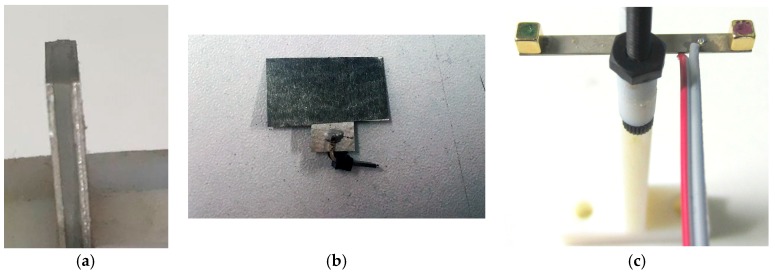
Images of fabricated test structures. (**a**) Galfenol-lead zirconate titanate (PZT) laminate. (**b**) Metglas-polyvinylidene fluoride (PVDF) ME laminate. (**c**) MME structure.

To build the Galfenol-PZT device, 25.4 mm diameter TdVib Galfenol (TdVib LLC, Ames, IA, USA) was cut using electrical discharge machining into two 10 mm × 20 mm × 370 µm sheets poled along the 20 mm length. The PE material used was 1.02 mm thick Piezo Systems PZT-5A (T140-A4E-602, Piezo.com, Division of Mide Technology, Woburn, MA, USA) which was cut using a diamond blade dicing saw to a single 10 mm × 20 mm sheet poled through the thickness. The three layers were then bonded together such that the PE layer was sandwiched between the ME layers. EPO-TEK H20S silver filled (conductive) epoxy was used to adhere the laminate. The epoxy was cured using a heat press, following the epoxy’s minimum cure instructions. Finally, two 0.635 mm right angle header pins were bonded to the top and bottom Galfenol. This bond was done using MG Chemicals silver conductive epoxy given that the joint wasn’t structural. This epoxy was cured overnight at room temperature. The resulting transducer can be seen in Figure 7a.

The Metglas-PVDF device was built in a fashion similar to that of the Galfenol-PZT device. Raw 23 µm thick 2605SA1 Metglas was cut using scissors into two 10 mm × 20 mm layers. The nature of amorphous Metglas is such that magnetostriction occurs at any orientation in the sheet plane so poling direction was unimportant. To match the very thin Metglas, metalized PVDF (TE 1-1004347-0) was used. These sheets themselves were a sandwich of 28 µm PVDF with 6µm silver ink electrodes on the top and the bottom, poled through the thickness. These sheets were also cut to 10 mm × 20 mm; however, a small tab was left so that electrical leads could be attached to the PE while using a non-conductive epoxy. In particular the nonconductive epoxy EPO-TEK H70E was used for its slightly thinner minimum bond line of less than 20 µm compared to the silver filled alternative which was measured on the Galfenol-PZT device to be about 35 µm. As before, the epoxy was cured in a heat press at the minimum prescribed cure. Similar leads were also bonded as before, however this time on the center flange of the PVDF. The final structure can be seen in Figure 7b.

The MME device was constructed from an of off-the-shelf PZT4A bimorph from Piezo Systems Inc. with overall dimensions of 32.55 × 3.175 × 0.38 mm. Two Neodymium magnet cubes of 3.175 mm on each edge were bonded to the ends of the PZT beam with cyanoacrylate. The final structure is shown in Figure 7c.

### 2.4. Experimental Methods

**Figure 8:**
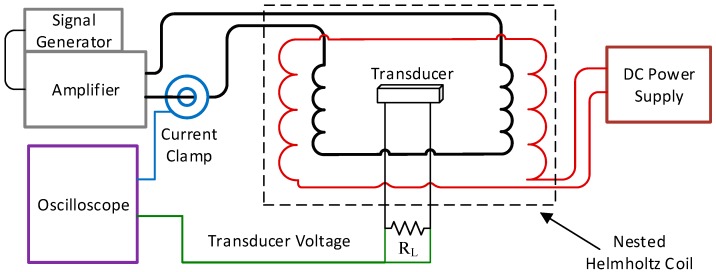
Magnetoelectric transducer experimental test setup diagram.

In order to characterize the ME and MME transducers, a nested Helmholtz coil was constructed to create a uniform alternating current (AC) magnetic field superimposed on a DC magnetic field. By superimposing the two fields, ME transducers could be both biased with the DC field and driven with the AC field. (Note that the MME transducer does not need a DC biasing field.). The system diagram for this setup and the nested Helmholtz coil are shown in Figure 8.

The nested Helmholtz coil can deliver an AC magnetic field ($H_{p}$) of 2-Oe (2 G in air) at 150 kHz with a 40-watt, 50 Ω amplifier (E&I 240L, Rochester, NY, USA) with no additional circuitry (i.e., tuned resonating capacitors) and a DC magnetic field ($H_{DC}$) of 16-Oe without exceeding the safe wire gauge current. The uniformity of the field was measured using an AlphaLab UHS2 gaussmeter. The AC coil was measured to have 2% field variation over ±1.5 cm at the coil origin (the point co-linear to the coil axis and equidistant from the inner coil faces) along the axial center line. The DC coil had less than 5% variation over the same length. For this and all other work the AC coil was driven by a Tektronix AFG1022 signal generator (Tektronix Inc., Beaverton, OR, USA) and either an E&I 240L (E&I, Rochester, NY, USA) or a Rigol PA1011 amplifier (Rigol Technologies Inc., Beaverton, OR, USA). The DC coils were driven with a B&K Precision 9201 power supply (B&K Precision Corporation, Yorba Linda, CA, USA).

**Figure 9:**
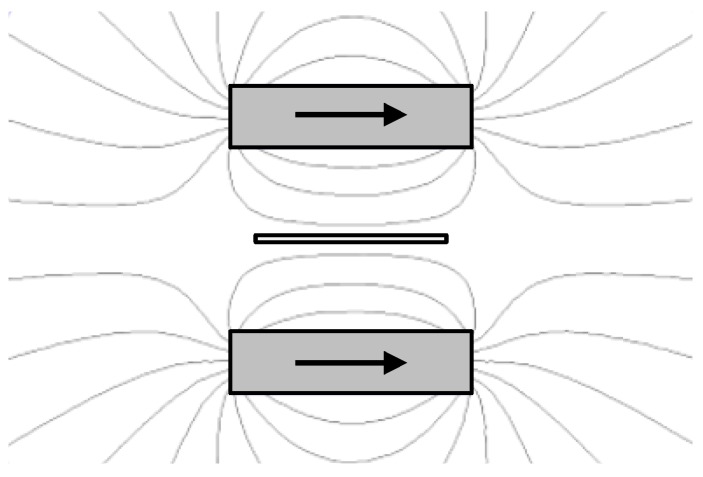
Biasing magnet arrangement and resulting field lines. Transducer is shown in between the two magnets.

The DC coil is sufficient to optimally bias the Metglas-PVDF ME structure. However, it is not sufficient to bias the Galfenol-PZT ME structure which requires a bias field of 100s of Oe. Therefore, a secondary biasing method was included in the test structure. Two parallel N52 Neodymium magnets were used as shown in Figure 9. By adjusting the distance between the two magnets with a 3D printed stage, the field seen by the centered transducer can be adjusted such that β=1 for the Galfenol-PZT ME structure.

The following three basic steps were performed to characterize the ME and MME devices and validate the lumped element models: optimize magnetic field bias, measure open circuit voltage as a function of frequency and magnetic field, measure power delivered to an optimized resistive load.

To determine the optimal field bias for each ME transducer, a small AC field at a frequency near the device resonance was applied while the DC field was slowly swept. The DC field that maximizes the open circuit AC voltage produced by the ME device was deemed to be the optimal field. The DC field was created by the DC coils for the Metglas-PVDF device and was found to be 22 Oe for the Metglas-PVDF device which is on the same order of magnitude as other reported Metglas transducers [25]. For the Galfenol-PZT device, the DC field was swept by varying the distance between the two permanent magnets. The DC bias field component parallel to the transducer length was measured along the length of the transducer with an Alphalab GM1-ST DC gauss meter yielding an average strength of 156 Oe with ±20 Oe deviation from average across the length of the device. Literature for bias field levels of Galfenol transducers is sparse, however for stiffer Terfenol-D transducers have reported bias fields of 200–500 Oe, depending on the structure design, so the value of 156 Oe seems reasonable [13,27].

Open circuit measurements were performed by sweeping the frequency of the magnetic field from 50 to 150 kHz at a rate of 12.5 kHz/s (10 s total duration). The field amplitude was set at $H_{p}=$ 1 Oe at 50 kHz, however this value attenuated as the sweep progressed due to the increasing coil impedance. To compensate, the magnetic field level and open circuit transducer voltage were measured simultaneously and then normalized for all of the sweeps performed. The normalization was done by performing an FFT on the signals then dividing the resulting transducer voltage amplitude by the field amplitude. The result was then multiplied by $H_{RMS}=$ 0.707 Oe to find the open circuit RMS voltage, $V_{ORMS}$, across the sweep frequency. It should be noted that this normalization does make the assumption that the transducer performance is linear, as does the model to which it will be compared. This assumption is common for many transducers and was validated experimentally for the ME transducer design by Bian et al. [28].

**Figure 10:**
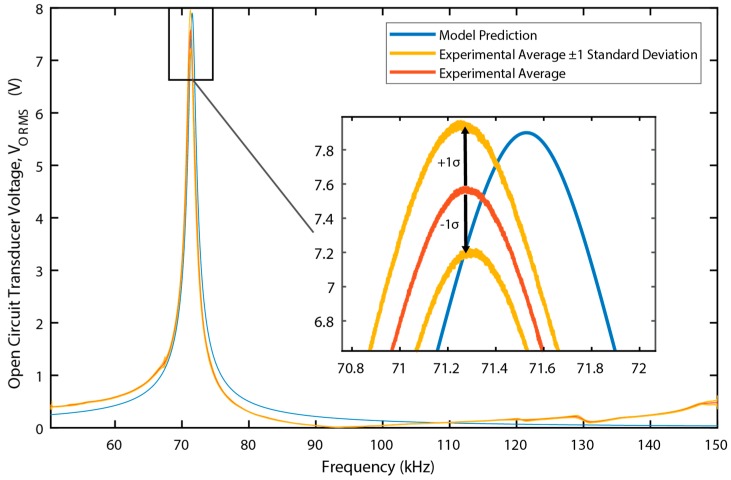
Repeated open circuit voltage vs frequency. Experimental Average, upper and lower deviation, and model prediction shown.

To ensure that test results were consistent, a repeatability test was performed with the Galfenol-PZT device. Nine tests were run. After each sweep, the stage holding the transducer was removed then replaced; after every 3rd sweep, the transducer was also removed from the bias structure entirely then re-clamped. The results of these sweeps can be seen in Figure 10, which indicate consistent performance.

Once the resonance frequency was determined, the voltage output was measured across a load resistor to determine the power generated. The load resistance was swept to experimentally determine the optimal load. The maximum generated power was taken to be the power dissipated at resonance through an optimal load resistor. This procedure was repeated for each of the three types of devices in order to validate the lumped element models.

## 3. Results

A primary goal of this work is to rigorously compare different magnetoelectric transducer architectures for use as wireless power receivers for biomedical implants. The approach taken is to develop models for each architecture, experimentally validate those models using off-the-shelf materials, and then use the models to optimize and compare each architecture within the constraints of a biomedical implant. This section contains both the experimental results that validate the models and the results of the constrained optimization procedure to compare each architecture.

### 3.1. Model Validation Results

**Figure 11:**
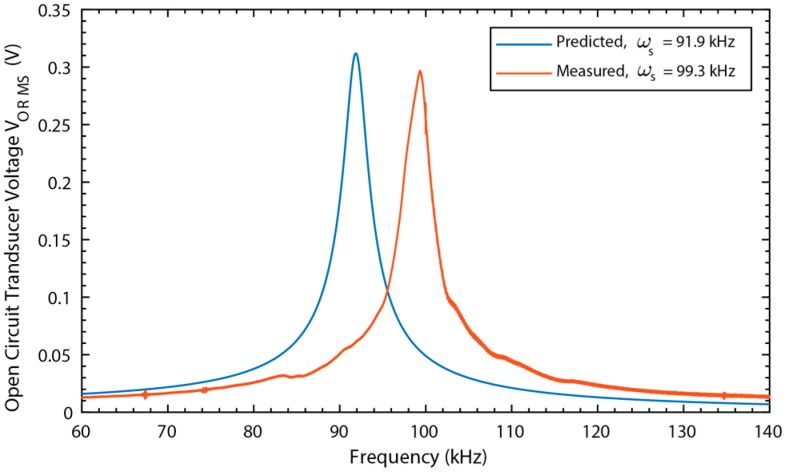
Metglas-PVDF open circuit voltage vs. frequency.

The measured and simulated open circuit voltage as a function of frequency for the Metglas-PVDF device is shown in Figure 11. (The open circuit voltage for the Galfenol-PZT device is shown above in Figure 10) In both cases, the DC magnetic field was optimally biased prior to the measurements. In the case of the galfenol-PZT device, both the measured voltage magnitude and resonance frequency match the simulation very closely. In the case of the Metglas-PVDF device, the measured peak output voltage is approximately 3% below the simulated value and the measured resonance frequency is approximately 8% higher than the predicted value. For this device, the epoxy layers between device structural layers is a significant fraction (20–35%) of the total laminate thickness. The model does not account for the stiffness and inertial effects of the epoxy layers. The model also assumes perfect strain transfer between layers which will introduce some errors given the relative thickness of the epoxy layers.

**Figure 12:**
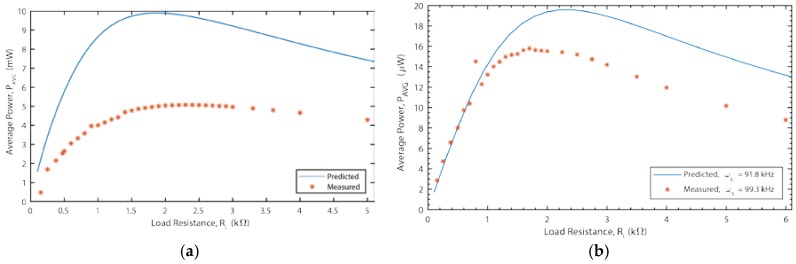
(**a**) Power output vs. load resistance for Galfenol-PZT device operating at 70.7 kHz. (**b**) Power output vs. load resistance for Metglas-PVDF device operating at 99.3 kHz.

Figure 12 shows the simulated and measured power output for the two ME devices versus load resistance at resonance. Clearly, the discrepancy between the model and devices increases when the resistive load is added for both ME devices. However, the discrepancy is worse (50%) for the galfenol-PZT device than for the Metglas-PVDF device (20%). This fact leads us to believe that the primary, although certainly not only, source of discrepancy are imperfections in the experimental setup. For example, the DC magnetic field bias mechanisms are different for the two devices. The fact that the galfenol-PZT device needs a larger bias field, which is applied by permanent magnets, means that the bias field is less uniform and less precisely controlled. Also, the effect of the mechanical mounting clamp will be different for the two devices. Further investigation and refinement in the experimental system is necessary to further investigate this discrepancy. Nonetheless, the model predicts the basic trends and there are reasonable explanations for the discrepancy. Therefore, it was felt that the model was sufficient to be used in a comparative optimization study.

**Figure 13:**
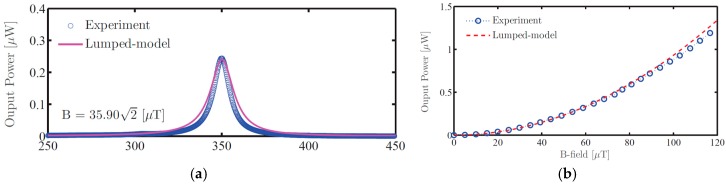
(**a**) Experimental and modeled MME transducer power output across frequency. (**b**) Experimental and modeled MME transducer power output under varying magnetic field at 350 Hz operating frequency. (Reproduced from [18], with permission from © 2018 IOP Publishing.).

Figure 13, which is reproduced with permission from [18], shows the power output of the MME device as a function of both frequency and AC magnetic field. In the case of the MME device, the equivalent circuit model matches the experimental output very closely. Given the fact that the MME device does not require a DC magnetic field bias and therefore is not affected by the strong nonlinearity in the voltage coefficient with respect to that bias nor the non-uniformity of that bias, this better agreement with experimental results is expected.

### 3.2. Comparative Analysis Results

Having established confidence in the basic design relationships encoded in the analytical models for the ME and MME architectures, a constrained nonlinear optimization procedure was used to compare transducer architectures given practical constraints for IMD devices. The goal of the optimization routine is to find the optimal ME and MME transducer designs within realistic constraints and compare the two. The lumped element models were implemented in MATLAB and MATLAB’s interior point optimization routines were used to find optimal solutions.

**Figure 14:**
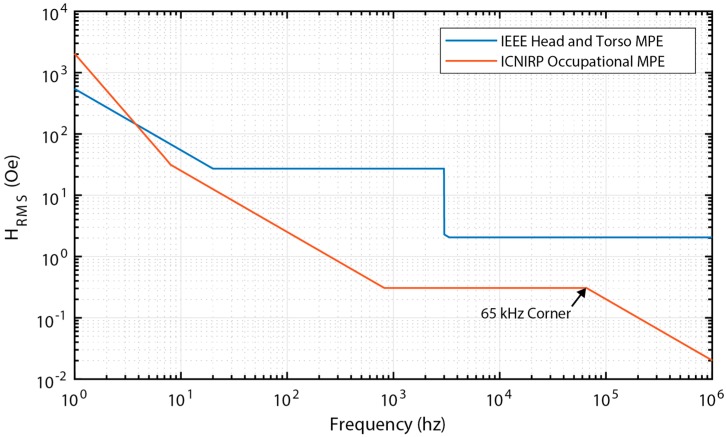
Allowable magnetic MPE levels for IEEE and ICNIRP standards. Adapted from [22,23,24].

Three basic types of constraints were placed on the optimization: volume, AC magnetic field amplitude, and geometric constraints to ensure manufacturability. The overall device volume was limited to 2 mm^3^. This constraint may seem somewhat arbitrary, but is meant to ensure applicability for minimally traumatic IMDs. The exact value of this constraint does not actually significantly alter the comparison results as long as the maximum size is on the order of 1–10 cubic millimeters. The maximum allowable magnetic field was determined using the IEEE standard on magnetic maximum permissible exposure (MPE) for the head and torso under controlled environmental conditions [22], [23], and the International Commission on Non-Ionizing Radiation Protection’s (ICNIRP) standard on maximum occupational exposure to magnetic fields [24]. Under both standards, the allowable MPE varies by frequency as shown in Figure 14. The ICNIRP standard is generally more conservative than the IEEE standard. Optimizations were performed separately using each standard. Finally, in most cases geometry constraints were coded as aspect ratio constraints to ensure reasonable device geometries for manufacture. A maximum aspect ratio of limit of 200:1 was set for the ratio of beam length ($l_{0}$) to total beam thickness ($t_{t}$) and for the ratio of beam width (w) to thickness ($t_{t}$). A maximum aspect ratio limit of 10:1 and a minimum aspect ratio of 0.1:1 were set for the ratio of beam length ($l_{0}$) to width (w). It should be noted that in both the aspect ratios, width refers to the entire structure width. However, the aspect ratios’ length, $l_{0}$ does not refer to the total structure length, but the length from both transducers’ center anchors to the free edges. This means for the ME transducer $l_{0}=0.5l$ and for the MME transducer $l_{0}=L$. Like the maximum volume constraint, these values are somewhat arbitrary based on the authors’ own experience. However, they do serve to keep device dimensions to values that could be manufactured and provide a reasonable basis for comparison of architectures.

For the ME device only, a minimum limit of 10:1 was placed on the length ($l_{0}$) to thickness ($t_{t}$) ratio. The reason for this constraint is that the equivalent circuit model begins to break down as the thickness approaches the same order of magnitude as the length. The model assumes that the extensional strain is uniformly transferred from the MS material through the thickness of the PE material. If the thickness gets large, this assumption breaks down as the strain is not uniform through the thickness of the PE material. The specific value of 10:1 was determined through finite element studies.

For the MME device only, a minimum limit of 1:4 was placed on the ratio of the magnet length ($L_{m}$) to beam length ($l_{0}$). If the length of the magnet becomes to large compared to the length of the beam, the beam bending model used loses accuracy. Finally, it should be noted that the optimization always chooses a substrate thickness ($t_{s}$) of zero meaning that the beam is a bimorph made entirely of piezoelectric material. Although this may not be the most practical implementation to ensure reliability, it does provide a bound on the maximum producible power. Finally a practical upper bound of 5 mm was placed on the magnet thickness (h).

The ME optimization was performed over the following six variables: transducer length (l), width (w), PE thickness ($t_{p}$), MS thickness ($t_{m}$) loaded natural frequency (ω), and load resistance ($R_{l}$). Two material configurations were optimized: Galfenol-PZT and Metglas-PZT using the material properties shown it Table 3 and Table 4. The mechanical quality factor ($Q_{m}$) was set to a value of 48, which was the average of the measured experimental values. Each optimization was performed under three different magnetic field constraints: the ICNRIP standard, the IEEE standard, and a baseline magnetic field of 1 Oe peak at any frequency. The results of the optimization are shown in Table 5.

The MME optimization was performed over the following five variables: beam length (or half transducer length) (L), beam width (w), piezoelectric thickness ($t_{p}$), magnet length ($L_{m}$), and magnet height (h). Both the operating frequency (ω) and load resistance ($R_{l}$) were calculated with closed form solutions as the six optimization parameters completely determine these two parameters. The PZT material was assumed to be PZT-5A with the same properties as in Table 3. The magnet was assumed to be Neodymium N52 with a remanant polarization of 1.46 Tesla and a density of 7500 kg/m^3^. As with the ME devices, the optimization was performed with the ICNIRP standard, IEEE standard, and a 1 Oe peak limitation at any frequency. The mechanical quality factor ($Q_{m}$) was set to 42 based on experimental results. The results of the optimization are shown in Table 5.

## 4. Discussion

Consider the two material sets used for the ME optimization (see Table 5). For all 3 magnetic field constraint conditions, the Metglas-PZT system outperforms the Galfenol-PZT system. Metglas has a higher magnetostrictive coefficient and is stiffer than Galfenol. This difference allows for greater device extension and a thicker piezoelectric layer thickness relative to the magnetostrictive layer. In addition, Metglas requires a much lower DC bias field, which makes it the clear choice for this application.

The IEEE and ICNIRP standards lead to very different optimal ME beam geometries. The IEEE standard leads to a short, wide, and thick structure ($l_{0}=1\;\mathrm{mm},\;w=10\;\mathrm{mm},\;t_{t}=0.1\;\mathrm{mm}$ for Metglas-PZT). From 3.35 kHz to 3 MHz, the magnetic field allowed by the IEEE standard is constant. As power generation will scale with the operational frequency and square of the magnetic field amplitude, the optimization will naturally try to maximize the resonance frequency of the ME structure. The optimization routine also selects a design that maximizes the transducer volume as would be expected. Therefore, as mass is more or less constant, the remaining opportunity for increased power is to increase stiffness within allowable constraints resulting in a short/wide beam. The power output results of this geometry (42.7 mW) are very promising.

As seen in Figure 14, the allowable magnetic field under the ICNIRP standard is constant from 0.82 to 65 kHz, above which point the allowable field decreases at a rate of approximately 20 dB per decade. Following the same scaling logic, one would expect the optimization routine to select a maximum volume design that operates at the 65 kHz discontinuity in the allowable field. In fact, the optimizer does select a design very close to this operating point (71.9 kHz). In order to maintain maximum volume within allowable geometric constraints, the resulting structure is a long and narrow structure ($l_{0}=12.6\;\mathrm{mm},\;w=1.26\;\mathrm{mm},\;t_{t}=0.063\;\mathrm{mm}$ for Metglas-PZT) in contrast to the structure constrained by the IEEE standard. The estimated power generated (62.6 μW) is enough for many wireless sensing applications, but certainly not as promising as the estimated power output resulting from the IEEE standard. Finally, the maximum material stresses generated for the ME device designs for each safety constraint were calculated and found to be at least an order of magnitude below the fracture stress (50–70 MPa [30]).

The MME architecture results in a structure with a far lower resonance frequency given that the transducer is excited in a bending vibration mode rather than in an extensional mode. Therefore, the MME device designs will generally be between 10’s of Hz and 1 kHz. Referring again to Figure 14, the allowable magnetic field under the IEEE standard for this frequency range is constant while the allowable field under the ICNIRP standard decreases at about 20 dB per decade. Therefore, the same scaling effects are at play with regard to the optimization algorithm. Under the IEEE standard constraint, the optimizer tends to shorten the beam to increase the resonance frequency. Under the ICNIRP standard constraint, the optimizer tends to lengthen the beam to increase the allowable magnetic field by decreasing the resonance frequency. A somewhat arbitrary height limit of 5 mm was placed on the magnet. In all cases, the optimization routine selects the maximum thickness magnet which increases the moment applied to the beam and therefore the stress and generated electric field in the PZT. Given this height constraint, the estimated power under the IEEE standard is about 5 times lower than the ME device (8.7 compared 42.7 mW). However, under the ICNIRP standard, the MME device estimated power actually goes up by about a factor of 2 (120 compared to 62.6 µW). This can be explained by the larger difference in allowable magnetic field as frequency decreases for the ICNIRP standard. However, two complicating factors should be discussed. First, the maximum stress in the PZT material for the MME device is 195 MPa and 37 MPa under the IEEE and ICNIRP standards, respectively. The fracture stress for PZT-5A is approximately 50–70 MPa. So, this MME-IEEE device would certainly fail. The MME-ICNIRP device would also be suspect given fatigue constraints. In order to reduce the stress generated, either the magnet height or the applied magnetic field needs to be reduced. Either of these options results in lower power output. Secondly, as previously discussed, the design that optimizes power output reduces the substrate thickness to zero meaning that bending beam is entirely made of piezoelectric material which is brittle. A thin piezoelectric beam with a large attached proof mass and no substrate between piezoelectric layers will almost certainly fracture in the presence of even a fairly mild shock. Therefore, to achieve a robust design a substrate needs to be added which would further reduce power output. Given these two complicating factors it appears that an ME architecture would almost always make a superior wireless power receiver.

## 5. Conclusions

The goal of this work was to investigate competing wireless power receiver concepts specifically applied to the size and safety constraints demanded for implantable medical devices (IMDs). Because the efficiency of traditional coil to coil wireless power transfer drops dramatically as size decreases, magnetoelectric (ME) and mechano-magnetoelectric (MME) receiver transducers were considered. Lumped element models were developed for each type of receiver that can be useful design aids in applications not only for IMDs, but also for wireless sensors and wearable sensors in general. These models were experimentally verified and subsequently used to produce optimized designs for an overall size constrain of 2 mm^3^. Two different safety standards, the IEEE [22,23] and ICNIRP [24], were used as constraints to the optimization process. The results of this study reveal that the ME architecture is definitely preferable under the IEEE standard and given practical constraints is also preferable under the ICNIRP standard, although in the latter case, the estimated power produced by each type of structure is similar. The optimized ME devices are estimated to produce 42.7 mW (21.35 mW/mm^3^) and 62.6 µW (31.3 µW/mm^3^) under the IEEE and ICNIRP standard, respectively. Although much work needs to be done to implement transducers of this size and performance level, these results are very promising in the context of being able to wirelessly power very small biomedical implants.

## Figures and Tables

**Table 1:** Lumped parameter equations for L-L magnetoelectric (ME) laminate. Adapted from [15].

Lumped Parameter Variable	Constitutive Equation
Characteristic Mechanical Impedance, $Z_{0}$	$Z_{0}=\rho_{avg}v*\left(A_{1}+A_{2}\right)\left[\frac{\mathrm{kg}}{s}\right]$
Average Laminate Density, $\rho_{avg}$	$\rho_{avg}=\frac{\rho_{ms\;}A_{2}+\rho_{me}A_{1}}{A_{1}+A_{2}}\left[\frac{\mathrm{kg}}{m^{3}}\right]$
Magnetoelectric Wave Speed, v	$v=\sqrt{\frac{n}{s_{33}^{H}}+\frac{\left(1-n\right)}{s_{11}^{D}}}\;\left[\frac{m}{s}\right]$
Volumetric Layer Ratio, n	$n=\frac{A_{2}}{A_{2}+A_{1}}$
Fundamental Frequency, $\omega_{s}$	$\omega_{s}=\frac{\pi v}{l}\;\left[\frac{rad}{s}\right]$
Effective Laminate Quality Factor, $Q_{m}$	$Q_{m}={\left(\frac{n}{Q_{ms}}+\frac{1-n}{Q_{me}}\right)}^{-1}$
Magnetostrictive Material Density, $\rho_{ms}$	Material property
Piezoelectric Material Density, $\rho_{pe}$	Material property
Magnetostrictive Quality Factor, $Q_{ms}$	Material property
Piezoelectric Quality Factor, $Q_{pe}$	Material property

**Table 2:** Lumped parameters for MME transducer.

Lumped Parameter Variable	Constitutive Equation
Bean Length, L	Dimension
Beam Length up to Magnet, $L_{0}$	Dimension
Beam Substrate Thickness, $t_{s}$	Dimension
PE Layer Thickness, $t_{p}$	Dimension
Magnet Mass, M	$M=\rho_{M}V_{M}\;\left[\mathrm{kg}\right]$
Magnet Mass, M	$M=\rho_{M}V_{m}\;\left[\mathrm{kg}\right]$
Beam Mass, $m_{b}$	$m_{b}=\rho_{s}V_{s}+\rho_{PE}V_{PE}\;\left[\mathrm{kg}\right]$
Equivalent Mass, m	$m=M+\frac{33}{140}m_{b}$ [m]
Equivalent Moment Force, $F_{m}$	$F_{M}=\frac{3M_{b}}{2l_{eff}}\left[N\right]$
Short-circuit Stiffness, $K_{0}$	$K_{0}=\frac{3{\left(YI\right)}_{c}}{l_{eff}^{3}}\;\left[\frac{N}{m}\right]$
Open-circuit Stiffness	$K_{1}=K_{0}+\Delta K\left[\frac{N}{m}\right]$
Piezoelectric Capacitance, $C_{0}$	$C_{0}=\frac{wL}{t_{p}\beta_{p}}\;\left[F\right]$
Open-circuit resonance Frequency, $\omega_{1}$	$\omega_{1}=\sqrt{\frac{K_{1}}{m}}\;\left[\frac{\mathrm{rad}}{s}\right]$

**Table 3:** Material properties used for Galfenol-PZT laminate model.

Property	Value
Piezo Systems PZT-5A4E	
Piezoelectric voltage coefficient, $g_{31,p}$	−11.6 × 10^-3^ Vm/N
Density, $\rho_{pe}$	7800 $\mathrm{kg}/m^{3}$
Piezoelectric compliance, $s_{11,p}$	15 × 10^−12^ $m^{2}/N$
Relative Dielectric constant, $K_{3}^{T}$ or ($1/(\beta_{p}\epsilon_{0})$)	1800
TdVib Galfenol	
Piezomagnetic coefficient, $d_{33,m}$	15–30 nm/A (15 used)
Density, $\rho_{ms}$	7800 kg/m
Magnetostrictive compliance, $s_{33}^{H}$	12.5–25.0 × 10^−12^ $m^{2}/N$ (16.7 used)

**Table 4:** Material properties used for Metglas-PVDF model [25,29].

Property	Value
TE Metallized PVDF	
Piezoelectric voltage coefficient, $g_{31,p}$	216 × 10^−3^ Vm/N
Density, $\rho_{pe}$	1780 $\mathrm{kg}/m^{3}$
Piezoelectric compliance, $s_{11,p}$	3.7 × 10^−10^ $m^{2}/N$
Relative Dielectric constant, $K_{3}^{T}$ or ($1/(\beta_{p}\epsilon_{0})$)	12
Metglas 2605SA1	
Piezomagnetic coefficient, $d_{33,m}$	25–50 nm/A (25 used)
Density, $\rho_{ms}$	7180 kg/m
Magnetostrictive compliance, $s_{33}^{H}$	9.09 × 10^−12^ $m^{2}/N$

**Table 5:** Optimization results.

Optimized Parameter	ME Galfenol-PZT		ME Metglas-PZT		MME PZT Bimorh	
	ICNIRP	IEEE	ICNIRP	IEEE	ICNIRP	IEEE
$l=2l_{0}$	21.5 mm	2 mm	25.2 mm	2 mm	NA	NA
$t_{P}$	15.9 µm	19.4 µm	18.1 µm	25.5 µm	10 µm	10 µm
$t_{m}$	33.5 µm	40.3 µm	22.4 µm	37.3 µm	NA	NA
w	1.1 mm	10 mm	1.26 mm	10 mm	0.4 mm	0.51 mm
$H_{p}$	0.44 Oe	2.89 Oe	0.40 Oe	2.89 Oe	5.56 Oe	38.39 Oe
2L	NA	NA	NA	NA	8.0 mm	8.0 mm
$L_{m}$	NA	NA	NA	NA	0.48 mm	0.39 mm
h	NA	NA	NA	NA	5.0 mm	5.0 mm
$\omega_{1}$	65 kHz	698 kHz	71.9 kHz	915 kHz	61 Hz	325 Hz
$P_{avg}$	15.6 µW	7.4 mW	62.6 µW	42.7 mW	120 µW	8.7 mW
